# Experimental and COSMO-RS Simulation Studies on the Effects of Polyatomic Anions on Clay Swelling

**DOI:** 10.1021/acsomega.1c03786

**Published:** 2021-09-28

**Authors:** Md Tauhidur Rahman, Berihun Mamo Negash, Alamin Idris, Mohammad Islam Miah, Kallol Biswas

**Affiliations:** ^†^Department of Petroleum Engineering, ^‡^Shale Gas Research Group (SGRG), and ^§^Department of Fundamental and Applied Sciences, Universiti Teknologi PETRONAS, 32610 Bandar Seri Iskandar, Perak Darul Ridzuan, Malaysia; ∥ Department of Engineering and Chemical Sciences, Karlstad University, 65188 Karlstad, Sweden; ⊥ Department of Petroleum and Mining Engineering, Chittagong University of Engineering and Technology, Chittagong 4349, Bangladesh

## Abstract

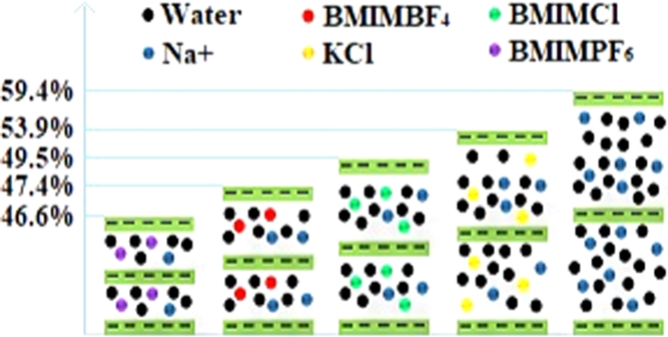

Ionic liquids (ILs) can play a vital role in clay swelling inhibition during hydraulic fracturing. Previous studies highlighted the effect of side-chain length attached to the cationic core and different anions possessing almost the same chemical properties on inhibition performance. However, polyatomic anions have the potential to superiorly inhibit swelling compared to monoatomic anions. In this study, three ILs, namely, 1-butyl-3-methylimidazolium chloride (BMIMCl), 1-butyl-3-methylimidazolium tetrafluoroborate (BMIMBF_4_), and 1-butyl-3-methylimidazolium hexafluorophosphate (BMIMPF_6_), were utilized to assess the effects of polyatomic anions on clay swelling inhibition. These performances were compared with the performances of a conventional inhibitor, potassium chloride (KCl). X-ray diffraction (XRD) testing was applied to check the mineral components present in the bentonite clay sample studied in this research. Clay swelling inhibition performance and rheological properties of these ILs were evaluated by the bentonite plate soaking test, linear swelling test, and rheological test. The swelling inhibition mechanisms were investigated through ζ-potential measurement, Fourier transform infrared (FT-IR) spectroscopy, and contact angle measurement. Moreover, COSMO-RS computer simulation was conducted to explain the inhibition mechanisms theoretically. The results demonstrated that BMIMPF_6_ showed superior inhibition performance and reduced the swelling by 21.55%, while only 9.26% reduction was attained by potassium chloride (KCl). The adsorption ability on the bentonite surface through electrostatic attraction, higher activity coefficient, and less electronegativity of PF_6_^–^ anion played a vital role in attaining such superior inhibition performance by BMIMPF_6_.

## 1 Introduction

The world continues to rely on hydrocarbons as a primary source of energy, while alternate and efficient sources of energy remain a mystery. Since most hydrocarbon energies are derived from conventional sources, these energy reserves are rapidly depleting.^1^ As a result, the massive unconventional energy sources have garnered new interest as a means of meeting the expanding energy demand. Unconventional reservoirs (such as shale gas, shale oil, gas hydrates, coal bed methane, and oil sands) vary from traditional reservoirs due to poor connectivity, low reservoir permeability, non-Darcy flow, low porosity, and the presence of high organic material.^2−4^ Due to the huge reserve volume of shale gas, it is a significant energy source among these unconventional reserves.^5^ These reservoirs consist of organic sediments, which exhibit ultralow permeability (10^–12^–10^–6^ μm^2^), narrow pore throats (diameters: 10^–3^–1 μm), and low porosity (approximately 4–12%).^6^ Traditional production methods are inadequate for producing hydrocarbons from these unconventional sources due to these constraints. Fortunately, emerging technologies such as hydraulic fracturing aid in the extraction of hydrocarbons from low-permeable shale formations.^7−9^

Hydraulic fracturing has become a key enabler for successfully extracting shale gas to meet the global energy demand. Hydraulic fracturing is the process of injecting fracking fluids (typically consisting of oil or water, chemical additives, and proppants) with a high pressure. The success of a fracking operation is dependent on not only the quantity and quality of induced fracture networks but also the aftereffects of the fracking fluids. Oil-based fluids are regarded efficient drilling/fracturing fluids due to their resistance to hydration and swelling, superior lubricity, borehole stability, high-temperature resistance, and corrosion inhibition qualities.^10^ However, the high initial cost, environmental hazards, operational safety, and disposal have limited the use of oil-based fluids during drilling and fracturing operations. On the other hand, water-based fluids are environmentally friendly, cost-effective, and capable of generating complex fracture networks. Despite their suitability for performance and economic production, water-based fluids have encountered certain challenges in shale formations. Shales are mostly composed of clay minerals (montmorillonite, illite, etc.), making them too water-sensitive. When the water-based fluids enter the shale formation, it causes shale instability due to hydration and swelling. Shale instability causes a number of issues, including solids building up in the mud, drill pipe being stuck, tight holes, plugging in the pores, and ultimately hole collapse, all of which are detrimental to shale gas output.^11−13^

Many additives, such as organic salts, inorganic salts, surfactants, polymers, amine derivatives, etc., were introduced as fracturing fluids to overcome the above-mentioned vital issues. They can inhibit the interaction of water and clay minerals, but their use is limited by certain constraints, such as lower inhibitive performance and environmental concerns.^14−16^

Over the years, the petroleum industry has been using mostly KCl as a clay swelling inhibitor at very high concentrations. KCl can give better performance by tightly fitting the interlayer spaces with potassium cations and combating osmotic pressure with chloride anions. However, it is required with a very high concentration for economic performance, which has arisen some challenges. Among the challenges, the environmental issues with a high concentration of KCl are the most concerning. According to the new regulations, disposal of any solution containing more than 3000 ppm chloride is restricted, while only 2 wt % KCl contains more than 9500 ppm chloride.^17^ As a result, there is an urgent need to find high-performance inhibitors with low environmental footprints.^18^

Recent approaches focus on bio-inhibitors^19^ and organic salts (ILs)^5^ to overcome environmental issues and improve inhibition performance. ILs are organic salts with lower melting points, which makes them different from molten salts.^20−22^ ILs are made up of three parts: an anionic core, a cationic core, and substituents attached to the cationic core.^23,24^ These three parts work differently and have significant effects on the clay swelling inhibition processes. The effects of alkyl chain length (substituents) attached to the imidazolium cation were investigated by Yang et al.^25^ Ahmed Khan et al. investigated various anions and discovered that halogen group anions have no significant effects because all halogen group anions have the same molecular properties.^26^ So far, there is no other substantial work on the impact of anions, especially polyatomic anions attached to imidazolium-based cations, on clay swelling inhibition. Based on the literature survey, it is found that a new study is required to investigate the above issues as well as to overcome the knowledge gaps in this area.

Therefore, in this study, the effects of 1-butyl-3-methylimidazolium chloride (BMIMCl), 1-butyl-3-methylimidazolium tetrafluoroborate (BMIMBF_4_), and 1-butyl-3-methylimidazolium hexafluorophosphate (BMIMPF_6_) on clay swelling inhibition have been investigated systematically. These three anions are different in their properties, such as their electronegativity, structure, size, etc. To evaluate the inhibition performance of these ILs, they were compared with the conventional inhibitor, KCl. Seven experimental techniques (bentonite plate soaking test, linear swelling test, rheology test, ζ-potential measurement, X-ray diffraction (XRD), Fourier transform infrared (FT-IR) spectroscopy, and contact angle measurement) were used in this study to evaluate the performance, investigate the effects of studied ILs on clay swelling inhibition, and explain the inhibition mechanisms of the studied ILs. Finally, the COSMO-RS simulation was performed to describe the inhibition mechanisms theoretically. In addition, some challenges to ILs are presented and the role of polyatomic anions on clay swelling inhibition is proposed. The results of this study will pave the way for the design of a high-performance clay swelling inhibitor for fracturing shale formations.

## 2 Results and Discussion

### 2.1 XRD Analysis

The mineralogical composition of the bentonite sample used for linear swelling, bentonite plate soaking, rheology, ζ-potential test, FT-IR analysis, and contact angle measurement was determined by XRD analysis. Table 1 shows the mineralogical composition of bentonite.

**Table 1:** Mineralogy of the Bentonite Powder Used in This Analysis

sample	mineral	composition	interlayer space
bentonite	montmorillonite (Si_7.80_Al_1.72_Cs_0.16_Fe_0.20_Mg_0.28_O_20_)	100 wt %	12.17 Å

Based on the XRD analysis, it was found that the bentonite used in this study was entirely composed of montmorillonite and the interlayer space was 12.27 Å. The swelling behavior of the shale is determined by the type and quantity of clays present in it. However, swelling clay minerals (smectite) are of importance because they allow water invasion into the interlayer spaces. Montmorillonite is a primary constituent of smectite, consisting of an octahedral sheet sandwiched between two tetrahedral sheets.^27^ This structure contributes to their proclivity toward swelling. Therefore, the bentonite powder fully composed of montmorillonite was selected for the experiments of this study.

### 2.2 Bentonite Plate Soaking Test

**Figure 1:**
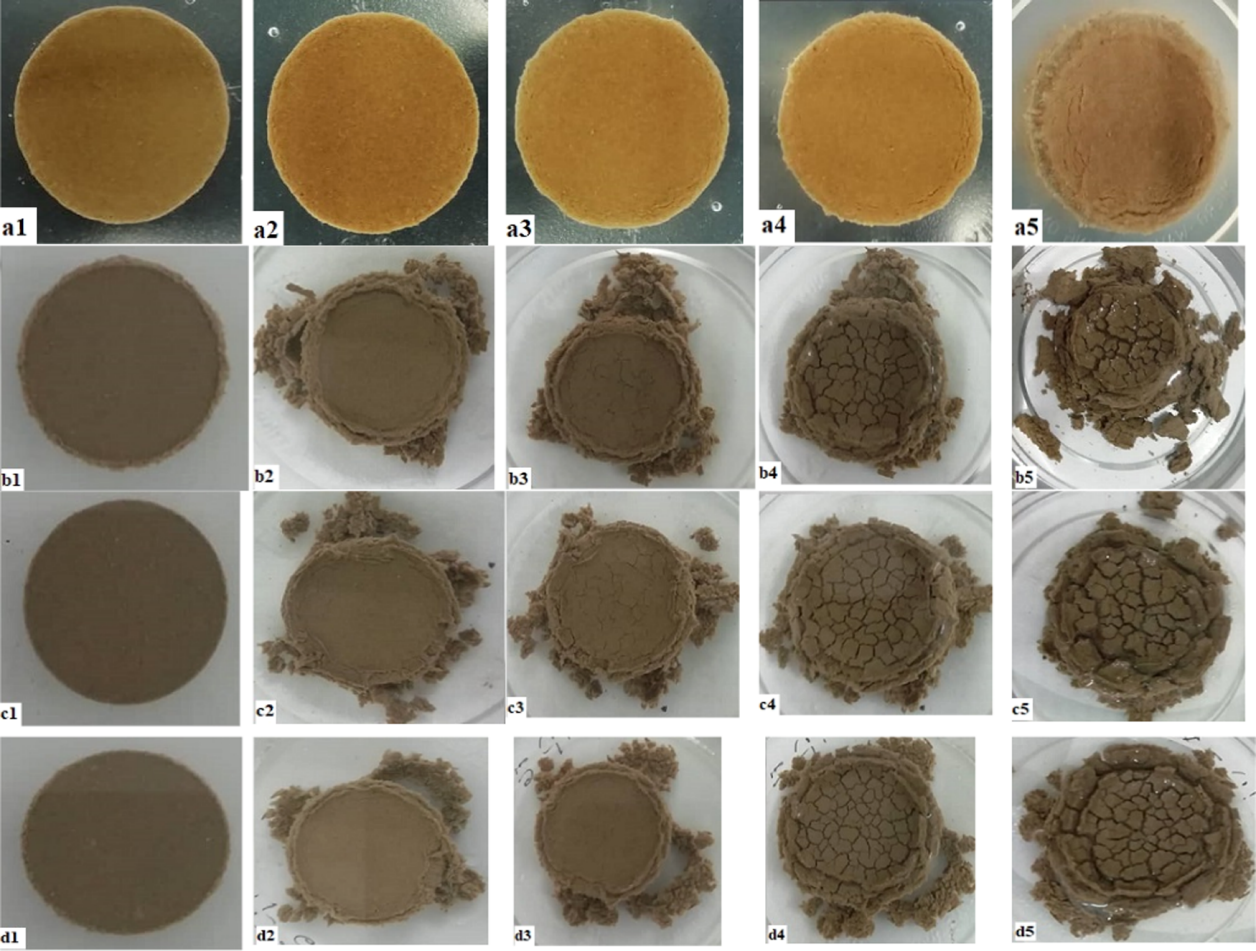
Morphological changes of bentonite plate soaked in various solutions: (a1–a5) deionized water; (b1–b5) BMIMCl; (c1–c5) BMIMBF_4_; and (d1–d5) BMIMPF_6_ after 0, 30, 60, 360, and 720 min, respectively.

During hydraulic fracturing operations, water-based fluids can enter the shale formation and result in clay hydration, eventually forming a collapse. This test was conducted to simulate water–clay interactions and observe their morphological changes. There were significant differences between the morphological changes of the bentonite plates soaked in water and water–inhibitor solutions. With passing time, the changes and differences become increasingly visible. In the case of deionized water, the bentonite plate became muddy. This phenomenon indicated the hydration potential of the bentonite when interacting with water.^12^ On the other side, when interacting with 2.0 wt % BMIMCl, BMIMBF_4_, and BMIMPF_6_, the bentonite plates split to a varying degree. These phenomena demonstrated the lower hydration potential of bentonite in IL solutions.^12^Figure 1 shows the morphological changes of the bentonite plate after being immersed in water and IL solutions. However, these morphological changes indicate the clay hydration reduction ability of the ILs but do not give any direct measure of hydration reduction amount.

### 2.3 Linear Swelling Test

**Figure 2:**
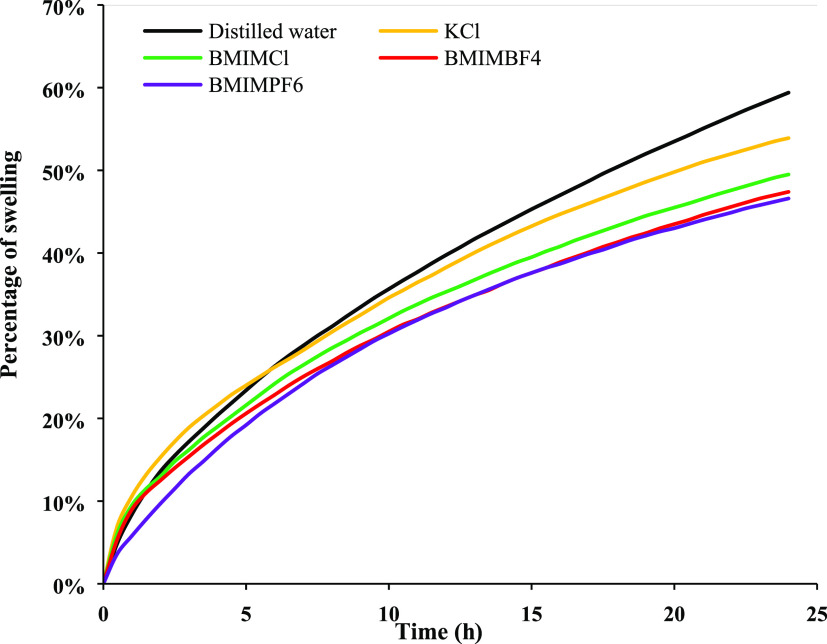
Swelling percentage for distilled water, KCl, BMIMCl, BMIMBF_4_, and BMIMPF_6_ for 24 h.

A linear swelling test can evaluate an inhibitor’s swelling inhibition efficiency as a function of time. Figure 2 depicts the swelling rate (expansion/increment rate) of bentonite wafers with various inhibitor solutions. For comparison and to check the inhibitors’ efficiency, freshwater (without any inhibitor) was also tested, and the results are demonstrated in Figure 2.

After 24 h, the bentonite wafer’s swelling rate in water was 59.40%, indicating an intense hydration and swelling tendency. When the water-based fluids were incorporated into the water-sensitive shale formation, water entered the interlayer space of clay minerals and hydrated the interlayer cations. The hydration of the interlayer cations weakened the bridging force between two neighboring clay sheets. It allowed a large volume of water to enter the interlayer space, causing swelling of the bentonite wafer.

When the studied ILs were added, the swelling of the bentonite wafer was decreased compared to water. The swelling reduction happened because of the adsorption of the ILs onto the bentonite surface through electrostatic attraction. This adsorption of the positively charged groups of ILs reduced the negative surface charge of bentonite, hence compressing the double electric layers. Some of the adsorbed IL molecules may be intercalated into the bentonite interlayer vacuum, where they may expel some water molecules. It reduced the water adsorption capacity of bentonite, which resulted in less hydration and swelling.

**Figure 3:**
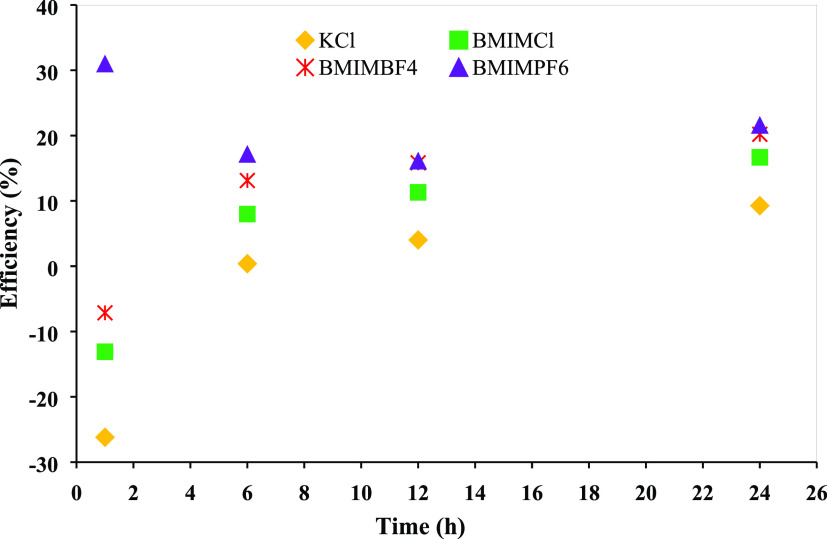
Swelling inhibition efficiency of different inhibitors at 1, 6, 12, and 24 h.

Figure 3 and Table 2 indicate that all of the ILs studied in this research showed less swelling tendency than water and better inhibition performance than the conventional inhibitor, KCl.

**Table 2:** Percentage of Swelling after 1, 6, 12, and 24 h with Various Inhibitors

experimental fluid system	swelling rate after 1 h (%)	swelling rate after 6 h (%)	swelling rate after 12 h (%)	swelling rate after 24 h (%)
water (100 wt %)	8.40	26.30	39.75	59.40
water (98 wt %) + KCl (2 wt %)	10.60	26.20	38.25	53.90
water (98 wt %) + BMIMCl (2 wt %)	9.50	24.20	35.30	49.50
water (98 wt %) + BMIMBF_4_ (2 wt %)	9.00	22.85	33.50	47.40
water (98 wt %) + BMIMPF_6_ (2 wt %)	5.80	21.80	33.40	46.60

After 24 h, the swelling rate was 53.90% for the fluids containing KCl, which indicated its inhibition potency. However, at the beginning (up to approximately 6 h), the expansion rate of the bentonite wafer immersed in KCl solution was higher than that immersed in pure water. A slight increase in swelling rate caused by KCl at the start is due to the penetration of clay minerals by the massive water volume during the cationic penetration and replacement processes.^28,29^ This also indicated a slower penetration rate of potassium ions than the cations of the ILs used in this study. Therefore, early, KCl promoted swelling more than pure water.

At the end of this experiment, the swelling rates were 49.50, 47.40, and 46.60% for BMIMCl, BMIMBF_4_, and BMIMPF_6_, respectively. With respect to the molecular weights, the sequence of these inhibitors is BMIMPF_6_ > BMIMBF_4_ > BMIMCl > KCl (Table 7). At 2 wt % concentration, their molar concentrations follow this ascending order; KCl > BMIMCl > BMIMBF_4_ > and BMIMPF_6_. This proved that a smaller number of ionic liquid molecules showed better inhibition performance than KCl. Figure 3 shows BMIMPF_6_ always showed better inhibition performance throughout the 24 h than the other inhibitors, and the efficiency sequence was BMIMPF_6_ > BMIMBF_4_ > BMIMCl > KCl. However, at the beginning, BMIMPF_6_ showed excellent inhibition efficiency than BMIMBF_4_ and BMIMCl. Over time, the efficiency of BMIMPF_6_ followed a decreasing trend while BMIMBF_4_ and BMIMCl followed an increasing trend, but BMIMPF_6_ showed superior performance over the 24 h. The difference in the performances of these three ILs was because of the anionic parts, as all three consist of the same cation (methylimidazolium) and substituent (butyl). Herein, the three anions are different in terms of their electronegativity and structure. The chloride anion is highly electronegative while the tetrafluoroborate anion is electronegative and the hexafluorophosphate is partially electronegative. The sequence of their electronegativity and size of their atomic structure are Cl^–^ > BF_4_^–^ > PF_6_^–^ and PF_6_ > BF_4_^–^ > Cl^–^, respectively. These differences eventually make differences in their performances.

**Figure 4:**
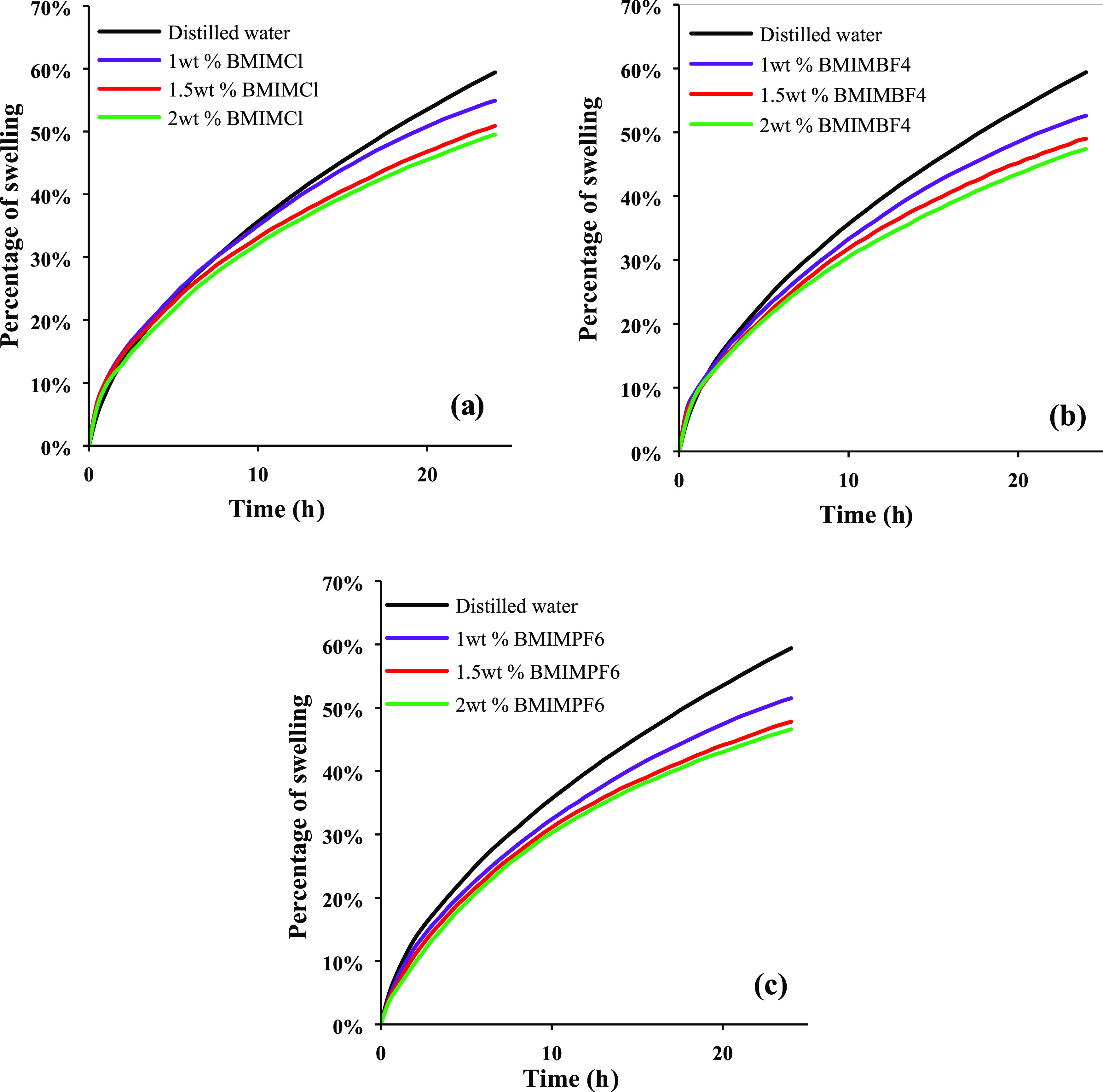
Swelling percentage for (a) BMIMCl, (b) BMIMBF_4_, and (c) BMIMPF_6_ at concentrations of 1.0, 1.5, and 2.0 wt % for 24 h.

Figure 4 illustrates the swelling rate of the bentonite wafer at 1.0, 1.5, and 2.0 wt % BMIMCl, BMIMBF_4_, and BMIMPF_6_. The swelling rate decreased considerably with increasing concentration of all ILs. The variation in the swelling rates between 1.5 and 2 wt % is quite little compared to 1 wt %. It is obvious that BMIMPF_6_ exhibited better swelling inhibition at all concentrations.

The swelling reduction efficiencies (compared to water) of BMIMCl, BMIMBF_4_, and BMIMPF_6_ at varied concentrations are shown in Table 3. At all concentrations, all of the ILs showed less swelling rate than water. BMIMPF_6_ performed admirably for swelling reduction even at low concentrations, outperforming the other ILs.

**Table 3:** Swelling Reduction Efficiencies (Compared with Water) of BMIMCl, BMIMBF_4_, and BMIMPF_6_ at Concentrations of 1.0, 1.5, and 2.0 wt %

	BMIMCl		BMIMBF_4_		BMIMPF_6_	
concentration (wt %)	swelling rate (%)	efficiency (%)	swelling rate (%)	efficiency (%)	swelling rate (%)	efficiency (%)
1.0	54.90	7.58	52.60	11.45	51.50	13.30
1.5	50.90	14.31	49.00	17.51	47.80	19.53
2	49.50	16.67	47.40	20.20	46.60	21.55

**Figure 5:**
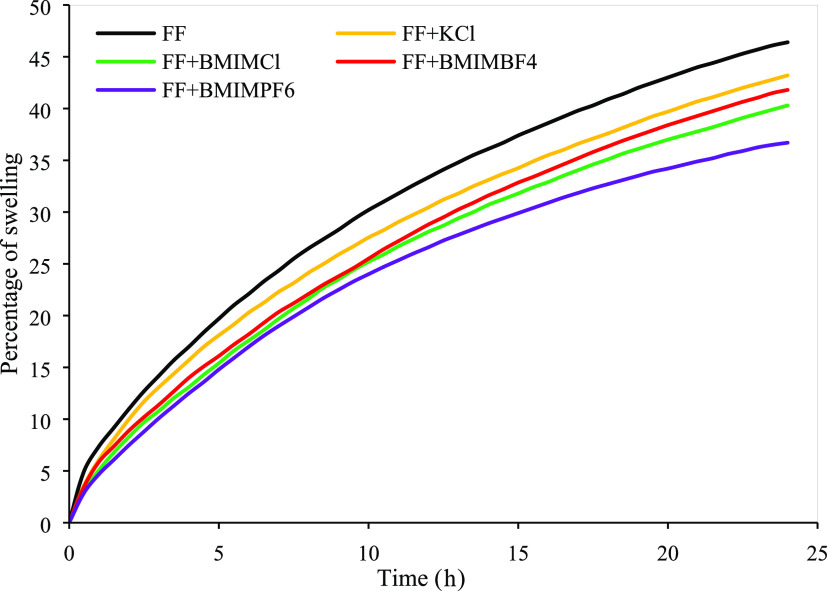
Swelling percentage for FF, FF+KCl, FF+BMIMCl, FF+BMIMBF_4_, and FF+BMIMPF_6_ for 24 h.

Figure 5 represents the percentage of swelling for FF, FF+KCl, FF+BMIMCl, FF+BMIMBF_4_, and FF+BMIMPF_6_ mixtures. The rate of swelling with FF after 24 h was 46.40%, which was reduced to 43.20, 40.30, 41.80, and 36.70% after adding KCl, BMIMCl, BMIMBF_4_, and BMIMPF_6_, respectively. BMIMPF_6_ retained its superior performance with FF in this case as well. However, with FF, BMIMCl showed better performance than BMIMBF_4_ and all of the ILs showed superior performance to KCl. These values indicate BMIMCl is more compatible with the FF than BMIMBF_4_. These results indicate that the studied ILs are compatible with the fracturing fluids.

### 2.4 Rheology Test

The rheological properties are analyzed to see the flow behavior of a drilling or fracturing fluid.^30^ However, rheological properties can also be used to assess a chemical’s ability to prevent clay swelling and dispersion.^5,31^ The fundamental understanding behind this principle is that the swelling and dispersion of clay minerals increase the viscosity and, as a result, the yield stress value.^32,33^ More swelling and dispersion enhance the number of clay particles in the (clay + water) fluid system, which increases the yield stress value of the fluid. Therefore, the yield stress/viscosity value can be a parameter to judge the swelling or dispersion inhibition efficiency of a chemical. A lower viscosity value is an indication of a chemical’s higher inhibition efficiency.

**Figure 6:**
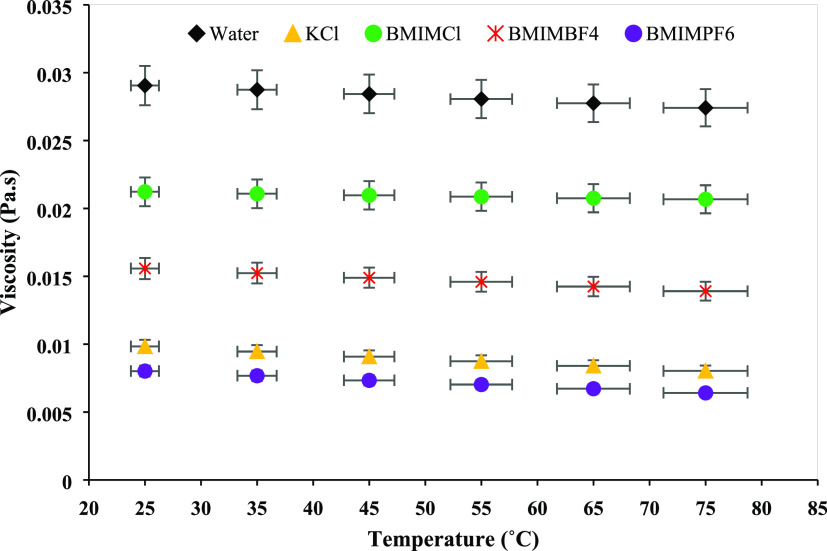
Temperature effects on the rheological properties of bentonite and bentonite–inhibitor suspensions.

The temperature effect on the rheological properties of the bentonite–water and bentonite–water–inhibitor suspensions is depicted in Figure 6. To test the temperature effects, the viscosity was calculated at a constant shear rate (1000 s^–1^) over a temperature range of 25–75 °C. The base fluid’s viscosity (water + bentonite) decreased at a rate of −3.13 × 10^–5^ Pa·s/°C. However, as the temperature rose, all of the ILs displayed a small decrease in viscosity. The viscosity degradation rates for BMIMCl, BMIMBF_4_, and BMIMPF_6_ were −1.06 × 10^–5^, −3.11 × 10^–5^, and −3.01 × 10^–5^ Pa·s/°C, respectively. This is due to the increment in the kinetic energy of fluids with increasing temperature. This higher kinetic energy means the higher molecular speed of the particles present in the system, which helps to overcome the resistance to flow, decreasing the viscosity at a higher temperature. The viscosity degradation for KCl was higher than the ILs, and the degradation rate was −3.44 × 10^–5^ Pa·s/°C. These results prove that the ILs studied in this research can work at a higher temperature.

**Figure 7:**
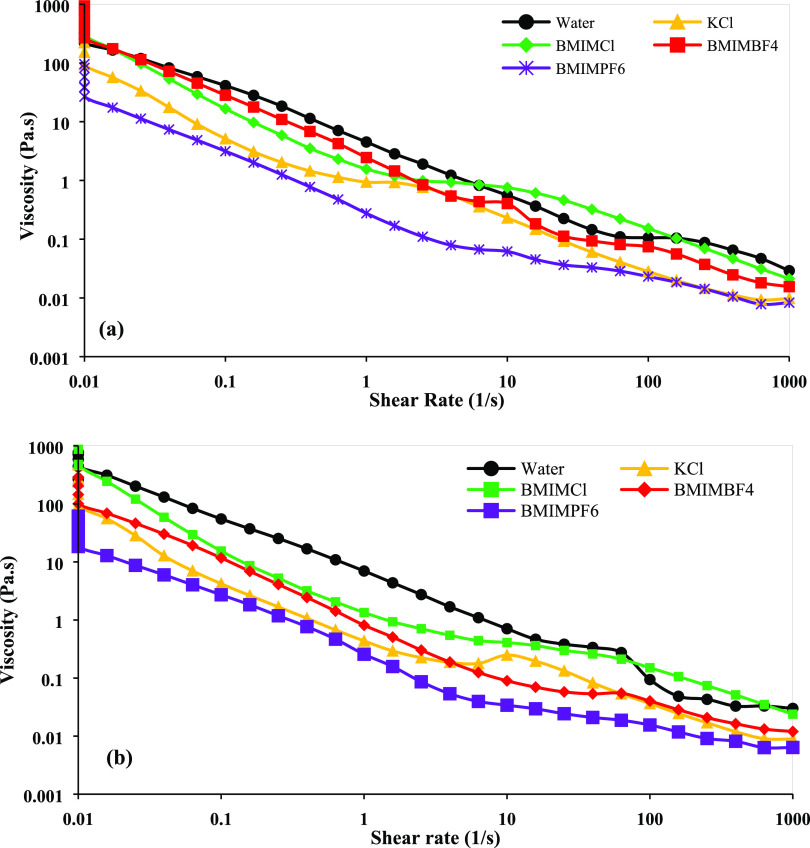
Flow behavior of bentonite–water and bentonite–water–inhibitor suspensions: (a) viscosity vs shear rate at 25 °C and (b) viscosity vs shear rate at 75 °C.

The viscosity is depicted in Figure 7, and Table 4 displays the rheological parameters of the bentonite–water and bentonite–water inhibitors (calculated by the Herschel–Bulkley model). The viscosity and yield stress of the bentonite–water suspensions reduced when the inhibitors were added. This is a positive sign for swelling and dispersion inhibition. After incorporating KCl, BMIMCl, BMIMBF_4_, and BMIMPF_6_, the yield stress of the bentonite–water suspension decreased to 1.2665, 2.9104, 2.8449, and 1.2256 Pa, respectively.

**Table 4:** Rheological Properties of Bentonite–Water/Bentonite–Water–Inhibitor Suspensions at 25 °C (Calculated by the Herschel–Bulkley Model)

inhibition system	yield stress, YS (Pa)	consistency index, *K* (Pa·s*^n^*)	flow index, *n*
water + BT	2.9443	0.0165	0.9798
KCl + water + BT	1.2665	0.0118	0.9475
BMIMCl + water + BT	2.9104	0.0168	0.9774
BMIMBF_4_ + water + BT	2.8449	0.0225	0.9358
BMIMPF_6_ + water + BT	1.2256	0.0117	0.9506

The reason behind these reductions is the interaction between the salt inhibitors and the bentonite clay present in the suspension. The ILs and KCl adsorbed on the surface of the bentonite, neutralized the negative surface charges, and gradually suppressed the double electric layers. Therefore, the repulsive force between the clay sheets is also reduced and prohibits swelling and dispersion. Among these inhibitors, BMIMPF_6_ exhibited the lowest viscosity, followed by KCl, BMIMBF_4_, and BMIMCl. This means BMIMPF_6_ is the best in terms of swelling inhibition but may create difficulties during proppant carrying facilities. On the other side, BMIMCl showed higher viscosity than the other inhibitors, which indicates that in the case of proppant carrying, BMIMCl is best, but for swelling inhibition, BMIMPF_6_ is superior. However, the proppant carrying issue can be solved using prehydrated bentonite. Prehydrated bentonite can retain the fracturing fluid’s viscosity high enough to carry proppants. In this experiment, nonhydrated dry bentonite powder was used to check the swelling inhibition efficiency of the ILs instead of proppant carrying capacity.

### 2.5 ζ-Potential

**Figure 8:**
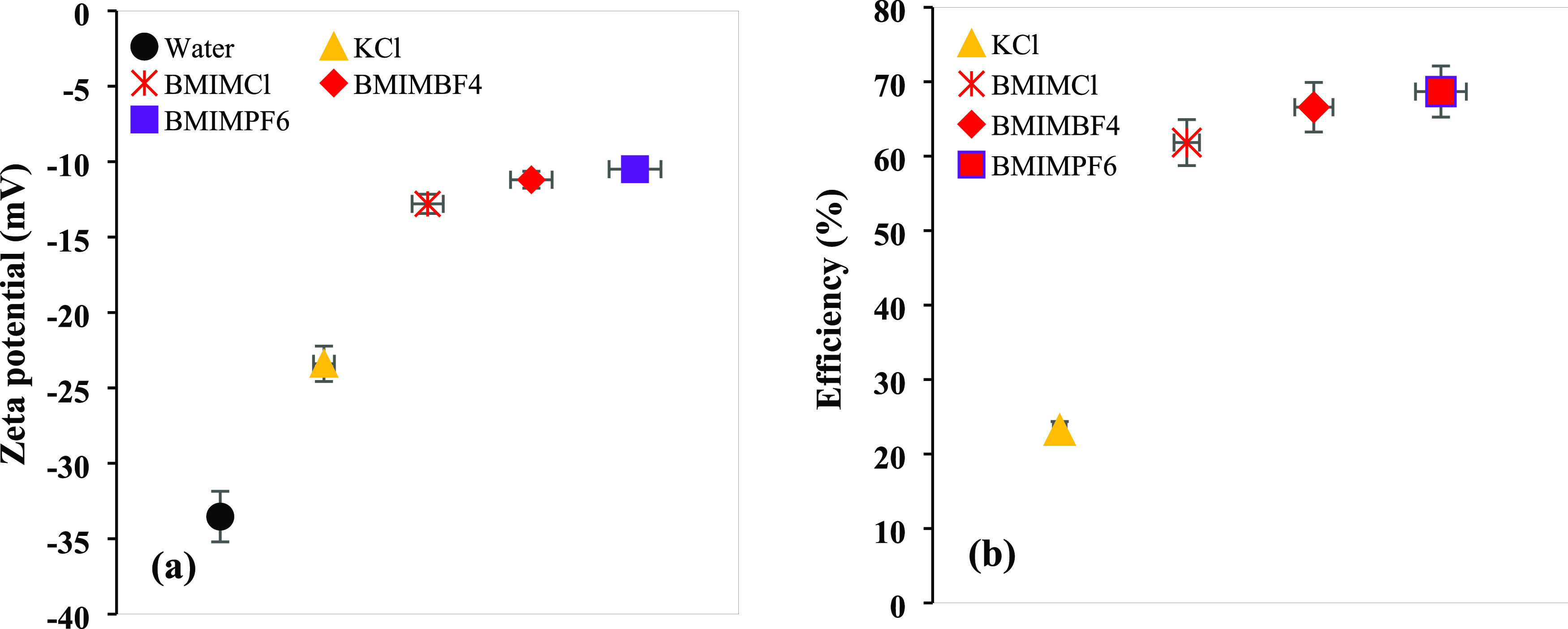
(a) ζ-Potential values and (b) efficiency of various inhibitors.

The linear swelling test results evaluate an inhibitor’s ability to prevent swelling. The ζ-potential value, on the other hand, is essential for analyzing inhibition mechanisms. Because of the isomorphic replacement, the surface of bentonite clay becomes negatively charged and has a strongly negative ζ-potential value.^34^ The lower absolute ζ-potential value is better for dispersion inhibition; on the other hand, the higher absolute value is better for colloidal system promotion.^35,36^ A potential inhibitor is described as one that can reduce the absolute ζ-potential value by 20%.^37^Figure 8a depicts the ζ-potential values of the bentonite suspensions in water and various inhibitor solutions.

The bentonite–water solution had a ζ-potential of −33.53 mV, suggesting a through dispersion and colloidal system. However, after incorporating the inhibitors, the values were −23.4, −12.8, −11.2, and −10.5 mV for KCl, BMIMCl, BMIMBF_4_, and BMIMPF_6_, respectively. These inhibitors proved that they demoted the colloidal system by inhibiting dispersion. Figure 8b shows that the ζ-potential reduction by KCl, BMIMCl, BMIMBF_4_, and BMIMPF_6_ was 23.21, 61.83, 66.58, and 68.68%, respectively. The negative ζ-potential value reduction by all of these inhibitors was more than 20% which means they should be considered as good inhibitors. These huge amounts of ζ-potential reduction were possible because of the reduction of bentonite surface charge. When these ILs entered the interlayer space, they were electrostatically attracted to the bentonite surface, and the positive sections of these ILs neutralized the negative surface charges. As a result, the double electric layers were reduced, which reduced the distance between two clay sheets and prevented clay dispersion. However, among these three ILs, the efficiency series was BMIMPF_6_ > BMIMBF_4_ > BMIMCl. The differences in their performance were due to the difference in the anionic part. Herein, PF_6_^–^ is partially electronegative while BF_4_^–^ and Cl^–^ are electronegative and strongly electronegative, respectively. The less electronegative anions help to reduce the ζ-potential value.

### 2.6 FT-IR Analysis

**Figure 9:**
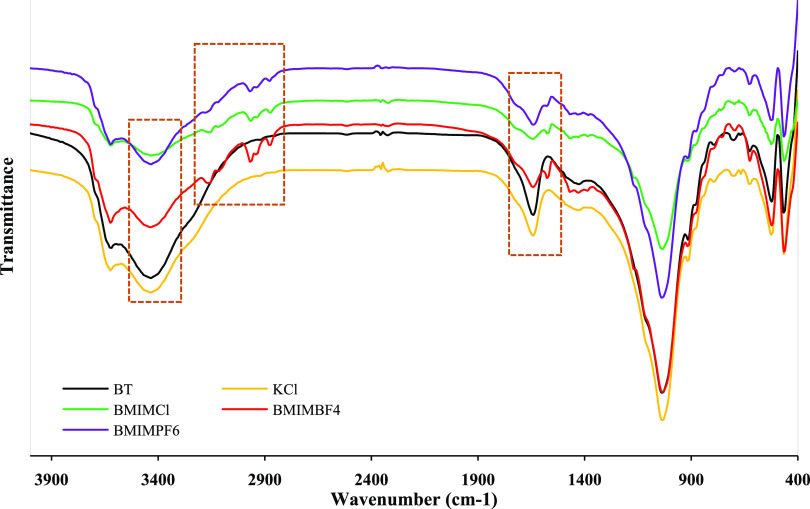
FT-IR spectra of bentonite, KCl–bentonite, BMIMCl–bentonite, BMIMBF_4_–bentonite, and BMIMPF_6_–bentonite composites.

To investigate the relationship between bentonite and inhibitors, FT-IR analyses were performed on bentonite, KCl–bentonite, BMIMCl–bentonite, BMIMBF_4_–bentonite, and BMIMPF_6_–bentonite composites. Figure 9 and Table 5 show the FT-IR spectra and some meaningful bonds present in bentonite, KCl–bentonite, BMIMCl–bentonite, BMIMBF_4_–bentonite, and BMIMPF_6_–bentonite composites, respectively.

**Table 5:** Significant Bonds Present in Bentonite and Inhibitor–Bentonite Composites^a^

peak	bond	BT	BT-KCl	BT-BMIMCl	BT-BMIMBF_4_	BT-BMIMPF_6_
521	Al–O–Si (D)	present	present	present	present	present
1036	Si–O (S)	present	present	present	present	present
1642	H–O–H (D)	DP	DP	SP	SP	SP
2874	C–H (S)	absent	absent	present	present	present
2970	C–H (S)	absent	absent	present	present	present
3435	ab. water (S)	DP	DP	SP	SP	SP
3621	O–H (S)	present	present	present	present	present

a D: deformation; S: stretching; DP: deep peak; SP: shallow peak.

The significant absorption peaks of the bentonite were as follows: the peak at 3621 cm^–1^ indicated the O–H stretching band, 3435 cm^–1^ indicated the stretching band of physisorbed water, 1642 cm^–1^ indicated the deformation of H–O–H, 1036 cm^–1^ indicated the stretching band Si–O, and 521 cm^–1^ indicated the deformation band of Al–O–Si. In the case of the IL–bentonite composite, new characteristic peaks were observed at 2970 and 2874 cm^–1^, indicating a C–H group stretching band. These two peaks prove the incorporation of ILs into the bentonite. The adsorption peaks at 1642 and 3435 cm^–1^ were stronger for bentonite while they were weaker for IL–bentonite composites. These weak peaks indicated the reduction of water molecules from the interlayer space of the bentonite. ILs, on the other hand, not only interacted with bentonite but also formed bonds with water. Therefore, it inhibited bentonite hydration and swelling to some extent by limiting the entry of water molecules into the bentonite interlayer space.

### 2.7 Contact Angle Measurement

Since shale is hydrophilic, it has a high sensitivity to water. A hydrophilic surface is quickly wetted by water molecules, while a hydrophobic surface is the opposite. When an inhibitor reaches the formation, it may change the surface of the clay from hydrophilic to hydrophobic or increase hydrophobicity. Therefore, determining the surface wettability shift is crucial for understanding the inhibition process of an inhibitor. Contact angle measurement is a simple way to assess wettability changes on a surface.

**Figure 10:**
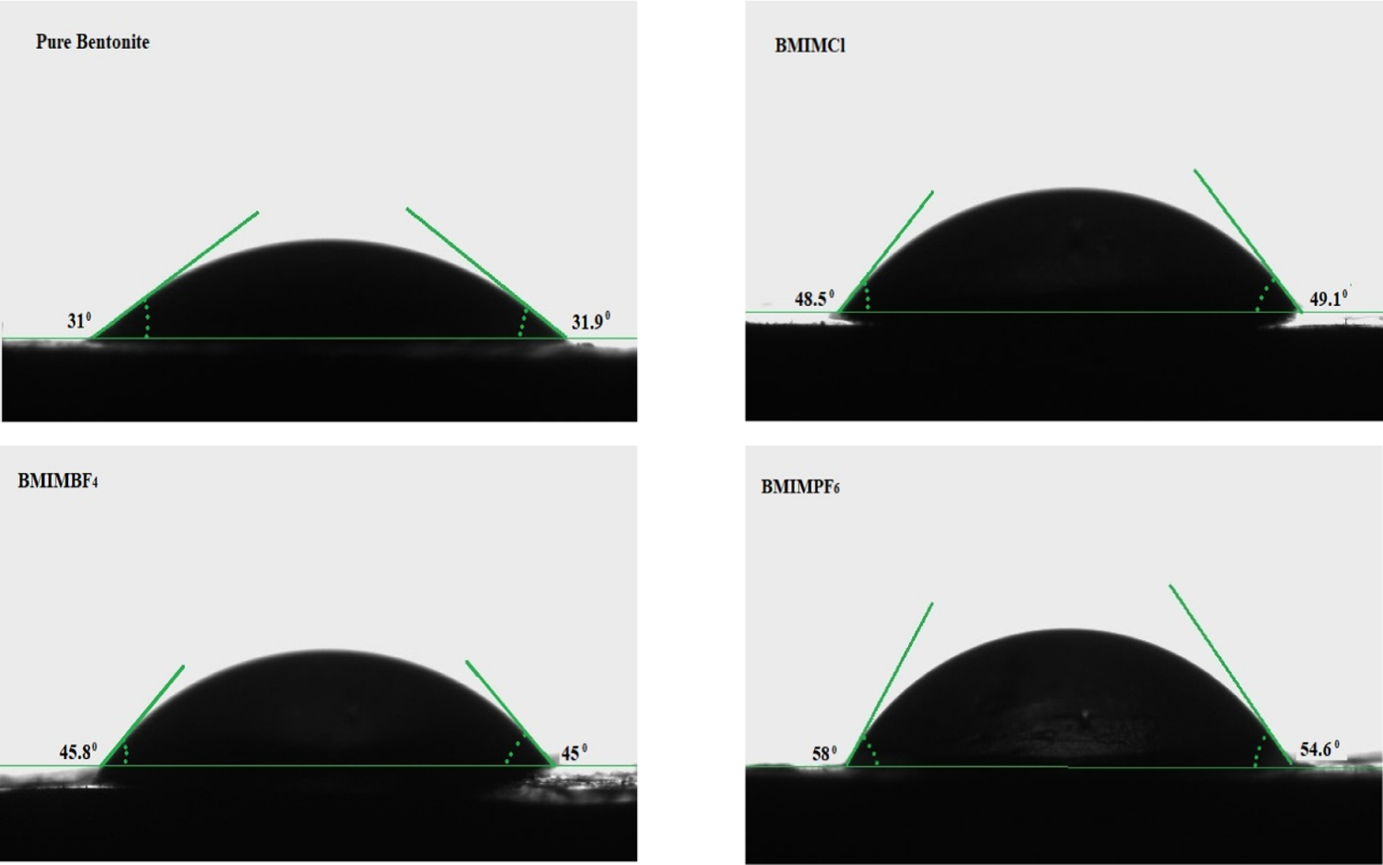
Contact angles between water drop and bentonite surface/bentonite surface modified with ILs.

Figure 10 depicts the contact angle between water drop and bentonite surface, as well as the contact angles between water drop and bentonite surface modified with ILs. The average contact angle between a water droplet and a pure bentonite surface was 31.45°, indicating that the bentonite surface is highly hydrophilic. An increase in the contact angle compared to a pure bentonite surface means that the bentonite surface is becoming more hydrophobic or less hydrophilic. Clay swelling inhibition benefits from higher hydrophobicity or lower hydrophilicity. Herein, the contact angles between the water droplet and the bentonite surface modified with BMIMCl, BMIMBF_4_, and BMIMPF_6_ were 48.8, 45.4, and 56.3°, respectively. These findings show that after ILs adsorb on the clay surface, the negative surface charge decreases, improving hydrophobicity and, as a result, reducing clay swelling.

### 2.8 COSMO-RS (Conductor-Like Screening Model for Real Solvents) Simulation

**Figure 11:**
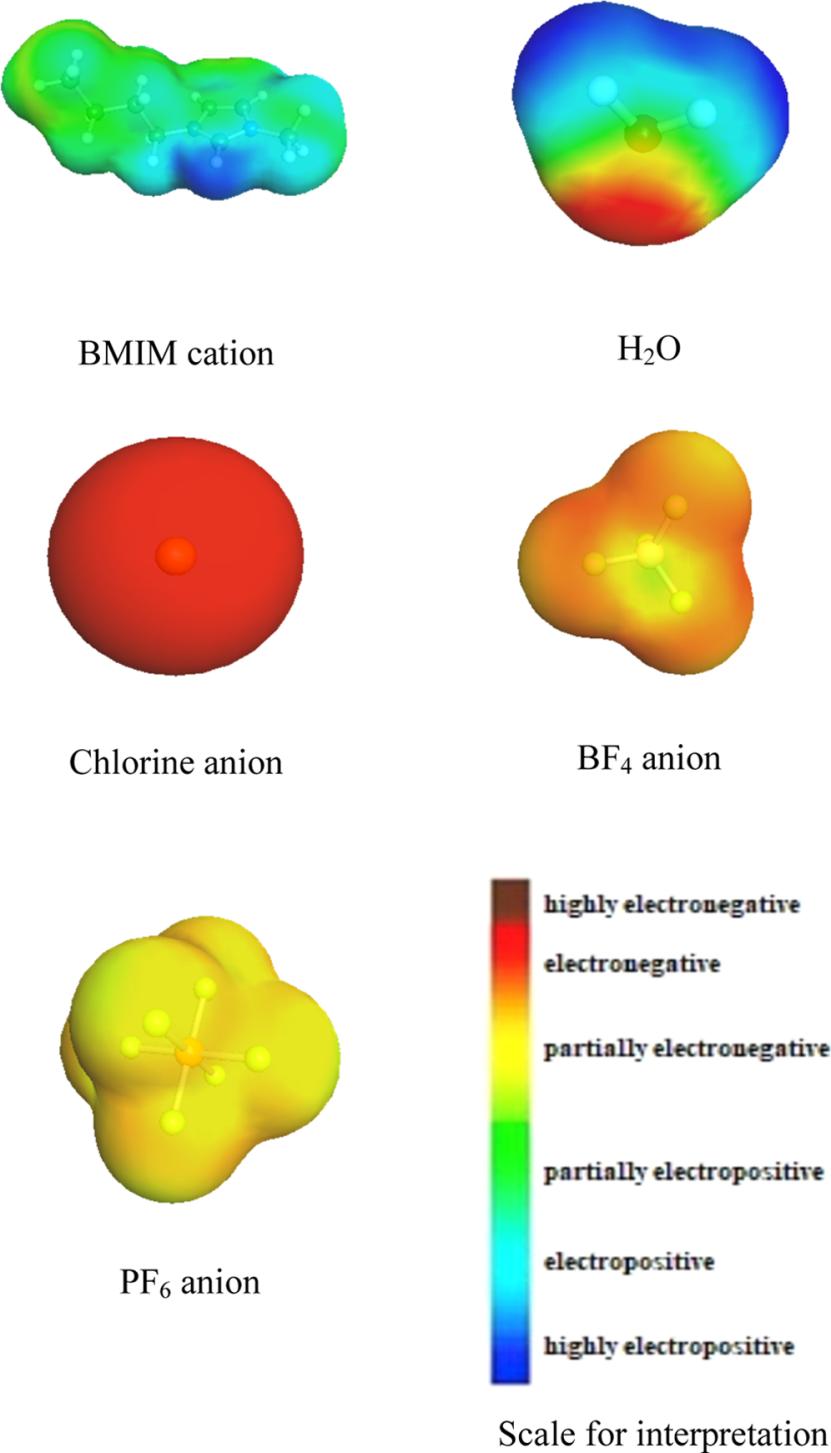
Sigma surfaces of the studied ILs (cations and anions) and water molecule.

The COSMO-RS simulation studies were conducted to gain a clear understanding of the effects of anions on the swelling inhibition processes. Herein, the sigma surfaces, activity coefficient, and hydrogen-bond energies were utilized to support the experimental results. Figure 11 represents the sigma surfaces of the cation (BMIM), anions (chloride, tetrafluoroborate, and hexafluorophosphate), and water molecules.

These sigma surfaces depict the charge distribution (electronegativity or electropositivity) of anions and cations, which is crucial for adsorption on the clay surface or interaction with water molecules in the interlayer spaces. The deep blue and deep red colors represent highly electropositive and highly electronegative charges on the chemical’s surface, respectively. On the surface of the BMIM cation, there is a deep blue region which indicates the presence of a highly positive charge in the cation. However, there are some large portions that are electropositive and partially electropositive. The positive charges help the BMIM cation to get adsorbed on the bentonite clay surfaces. On the other side, the anions used in this research are highly electronegative (Cl^–^), electronegative (BF_4_^–^), and partially electronegative (PF_6_^–^) (Figure 11). This difference in the surface charge directly affects the activity coefficient and hydrogen-bond formation capacity of the inhibitors, which eventually makes a difference in their swelling inhibition performance.

Table 6 shows the activity coefficient and hydrogen-bond energies for the studied ILs mixed with water. The activity sequence of the inhibitors according to the activity coefficient is BMIMPF_6_ > BMIMBF_4_ > BMIMCl. Following the activity coefficient, BMIMPF_6_ is more active than the other inhibitors. This higher activity of BMIMPF_6_ helped to show better inhibition performance from the beginning. Therefore, the swelling inhibition efficiency of this inhibitor was very high compared to the other inhibitors. The reason behind this higher activity coefficient is the low attraction force between the cation and anion present in BMIMPF_6_. Due to being partially electronegative, PF_6_^–^ is loosely attached to the imidazolium cation. On the other side, BF_4_^–^ (electronegative), and Cl^–^ (highly electronegative) anions feel more attraction force from the imidazolium cation. This less attraction force between the cation and anion makes a chemical more active. However, in the swelling test, BMIMCl promotes swelling at the beginning stage. That happens due to the lower activity of this inhibitor than the other inhibitors. The hydrogen-bond energy of these ILs with water molecules can be another parameter to assess their inhibition mechanisms. According to Table 6, the hydrogen-bond energies of BMIMCl, BMIMBF_4_, and BMIMPF_6_ with water molecules are −26.05303, −11.23145, and −3.63325 kcal/mol. Herein, the minus (−) sign represents energy production during the bond formation instead of representing negative numbers. These values indicate that BMIMPF_6_ creates less hydrogen bonds with water molecules while BMIMCl creates the highest number of hydrogen bonds. Though these three ILs have the same cation, their hydrogen-bond energies are different. Therefore, it is well understood that the differences in the hydrogen-bond energies come from their different anions. These values prove the anions also have an impact on hydrogen-bond formation with water molecules. Therefore, when the BMIMCl enters the interlayer space, it may create hydrogen bonds with more water molecules and facilitate water invasion into the interlayer space. On the other side, less hydrogen-bond energy between BMIMPF_6_ and water molecules reduced the water invasion into the interlayer space, hence reducing clay swelling.

**Table 6:** Studied Parameters for Different ILs Generated by COSMO-RS Computer Simulation

inhibition system	concentration	activity coefficient	*E*_HB_ (kcal/mol)	surface area (*A*^2^)	volume (*V*^3^)
BMIMCl + water	BMIMCl (2 wt %), water (98 wt %)	0.956145	–26.05303	255.04484	235.95007
BMIMBF_4_ + water	BMIMBF_4_ (2 wt %), water (98 wt %)	0.982126	–11.23145	292.80484	272.71322
BMIMPF_6_ + water	BMIMPF_6_ (2 wt %), water (98 wt %)	0.988334	–3.63325	316.71993	303.54156

### 2.9 Proposed Role of Polyatomic Anions in Clay Swelling Inhibition

ILs consist of two main constituents: a cationic core and an anionic core. In addition, the cationic core can be modified using a variety of substituents. When IL reaches the interlayer space, it is adsorbed by electrostatic attraction forces on the clay surfaces. Due to the adsorption on the clay surface, ILs neutralized the negative surface charges and inhibited the double electric layers, which are proven by the ζ-potential value. The neutralization of negative charges decreased the hydrophilicity of clay surfaces, resulting in a decrease in their attraction to water molecules. From the simulation and experimental data, it can be concluded that inhibitors with greater activity coefficients work better in terms of inhibition. As previously noted, less electronegative anions contributed to the increased activity of the ILs. This phenomenon occurs because less electronegative anions are loosely bonded to the cations. As a result, cations have a better interaction with bentonite surfaces that contain less electronegative anions. In addition, some less electronegative anions may increase hydrophobicity or decrease the hydrophilicity of the clay surface. This also aids in preventing water from entering the interlayer space. According to the simulation results, it appears that less electronegative anions (PF_6_^–^ and BF_4_^–^) reduce the hydrogen-bond formation between the ILs and water molecules. Due to the lower hydrogen-bond energy between the ILs and water molecules, water invasion into the interlayer space was reduced, resulting in less clay swelling.

### 2.10 Challenges of Ionic Liquids

The cost of ionic liquids is a significant impediment to their industrial application.^38^ However, they exhibit excellent inhibitory properties even at low concentrations. Thus, large production of ILs may ensure their economic viability for application in the hydraulic fracturing process. ILs are considered environmentally friendly due to their minimal vapor pressure. However, some studies expressed concern about the consequences of ILs on the ecosystem. While anionic moieties have a negligible environmental impact, the alkyl chain length connected to the cationic core has a significant impact on the environment.^39^ Shorter alkyl chains connected to cations were shown to be less harmful, but longer alkyl chains were found to be more toxic.^40−42^ By adding functional polar groups to the alkyl chain, the toxicity of the compound may be decreased.^39^ Further study may focus on cost-effective manufacturing and tailoring of environmentally favorable high-performance ILs.

## 3 Concluding Remarks

Three imidazolium-based ILs with different anions were studied to investigate the effects of anion on clay swelling inhibition processes during hydraulic fracturing operations. All of the ILs showed better inhibition performance compared to potassium chloride. The imidazolium cation quickly adsorbs onto the negatively charged bentonite surface, resulting in a reduction in the double electric layers. The butyl side chain, on the other hand, can obstruct water from entering the interlayer space through making a hydrophobic shield. The swelling inhibition efficiencies by BMIMPF_6_, BMIMBF_4_, and BMIMCl were 21.55, 20.20, and 16.67%, respectively. Despite the same cation and side-chain, these three ILs showed different inhibition performances because of the different anions. The COSMO-RS simulation study confirmed that the electronegativity and the surface charge distribution of these three anions are not the same. Cl^–^ is highly electronegative, BF_4_^–^ is electronegative, and PF_6_^–^ is partially electronegative. The less electronegative, PF_6_^–^ showed less attraction force with imidazolium cation, increasing the activity coefficient of BMIMPF_6_. The higher activity coefficient helped to reduce the swelling percentage from the beginning of the linear swelling test. Moreover, the less electronegative PF_6_^–^ anion helped to decrease the negative surface charges of the bentonite, which is also important for swelling inhibition. In addition, less electronegative anions (PF_6_^–^ and BF_4_^–^) formed less hydrogen bonds with the water molecules. Therefore, these anions may prevent further clay hydration by inhibiting water invasion into the interlayer space. Hence, a less electronegative anion is recommended for clay swelling inhibition processes.

## 4 Materials and Methods

### 4.1 Materials

In this work, three ILs with the same cation but different anions, BMIMCl, BMIMBF_4_, and BMIMPF_6_ were precisely studied. To study the swelling phenomenon, bentonite containing water-sensitive montmorillonite was used, which was obtained from SCOMI Oiltools. In addition, all of the ILs were procured from Avantis Laboratory Supply, Malaysia. Table 7 summarizes the physiochemical properties of the chemicals used in this work.

**Table 7:** Physiochemical Properties of the Chemicals Used in This Work^a^

chemical name	chemical formula	MW (g/mol)	purity	cation	anion	source
potassium chloride	KCl	74.55	AG	K^+^	Cl^–^	Avantis Laboratory Supply, Malaysia
BMIMCl	C_8_H_15_ClN_2_	174.67	≥98.0%	C_8_H_15_N_2_^+^	Cl^–^	Avantis Laboratory Supply, Malaysia
BMIMBF_4_	C_8_H_15_BF_4_N_2_	226.02	≥98.0%	C_8_H_15_N_2_^+^	BF_4_^–^	Avantis Laboratory Supply, Malaysia
BMIMPF_6_	C_8_H_15_PF_6_N_2_	284.18	≥97.0%	C_8_H_15_N_2_^+^	PF_6_^–^	Avantis Laboratory Supply, Malaysia

a AG: Analytical grade.

### 4.2 XRD Analysis

XRD analysis was conducted for characterization or mineralogical analysis of the bentonite sample used in this study. An amount of 11 g of bentonite powder as a representative sample was analyzed by an X’Pert^3^ powder X-ray diffractometer. Furthermore, Highscore (plus) software (PANalytical) was utilized to analyze the X-ray scans.

### 4.3 Bentonite Plate Soaking Test

The bentonite plate soaking test was used to investigate the hydration characteristics of the bentonite wafer through its morphological changes when exposed to water and inhibitor solutions. Clay hydration is a key reason behind clay swelling. For this experiment, the bentonite wafer was prepared by pouring 10 g of bentonite powder into a pressure chamber and continuously applying 1000 psi pressure for 30 min. Following that, the wafers were placed in glass beakers and 2.0 wt % BMIMCl, BMIMBF_4_, and BMIMPF_6_ solutions were added. To observe the physical changes, photographs of these wafers were taken after 0, 30, 60, 360, and 720 min, respectively. Table 8 represents different experimental environments for the bentonite plate soaking test.

**Table 8:** List of Fluids Used in the Bentonite Plate Soaking Test

	test fluids
bentonite wafer plate (10 g)	distilled water
	2.0 wt % BMIMCl
	2.0 wt % BMIMBF_4_
	2.0 wt % BMIMPF_6_

### 4.4 Linear Swelling Test

The linear expansion due to the water–clay interaction is investigated by this method, which takes a long time to interpret.^43^ In this test, the sample did not grind as it was already in powder form. First, 13 g of dry bentonite powder was put into a pressure chamber and compacted at a pressure of 1450 psi for 1 h. After compaction, the compacted bentonite wafer’s initial height and diameter were measured and placed into an automated dual-core HPHT linear swell meter (M46000). The wafers were then saturated with 75 mL of water or 1.0, 1.5, and 2.0 wt % water–inhibitor solutions, and the swelling phenomenon was monitored for 24 h under 1000 psi pressure and at room temperature. Also, a fracturing fluid (FF) was prepared by adding 0.40 wt % guar gum, 0.20 wt % potassium carbonate, 0.10 wt % borate, 0.20 wt % HPAM, and water. To check the compatibility of the used ILs with FF, 2 wt % IL was added to the FF and the swelling phenomenon was monitored. The swelling heights and percentages were recorded by data acquisition software, which was connected to a computer. The swelling percentage was calculated by the software utilizing the following equation
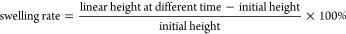
1Furthermore, to compare the swelling inhibition efficiencies of different ILs, the following equation was utilized.
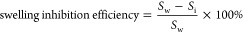
2where *S*_w_ represents the swelling height or percentage in water and *S*_i_ represents the swelling height or percentage in inhibitor’s solutions.

### 4.5 Rheology Test

The viscosity and yield stress were determined during rheology tests to investigate the flow behavior of bentonite–water and bentonite–water–inhibitor solutions. Moreover, the viscosity values were utilized to evaluate the swelling inhibition efficiencies of the studied ILs. The sample solutions for this experiment were prepared by adding 4 wt % bentonite and 2 wt % inhibitors. Then, the mixer of the bentonite–water–inhibitor was stirred for 30 min using a Fann five-spindle multimixer. The rheological properties of the sample solutions were analyzed using a Discovery Hybrid Rheometer (DHR-1). The data was collected for shear rates ranging from 0.01 to 1000 s^–1^ at 25 and 75 °C, respectively.

### 4.6 ζ-Potential Measurement

The samples for ζ-potential measurements were prepared by combining 0.2 wt % bentonite powder, 2 wt % IL, and water. First, 0.2 g of bentonite was mixed with 98.80 mL of water using a magnetic stirrer for 24 h. After that, 0.4 g of IL was added to 19.6 mL of the stirred solution and again magnetically stirred for 16 h. Finally, a ζ-potential analyzer (zeta-sizer Nano ZSP) was used to determine the ζ-potential value of water–bentonite and water–bentonite–inhibitor solutions. Furthermore, the efficiency of the inhibitors in reducing ζ-potential value compared to water was determined by the following equation.
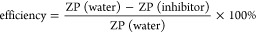
3where ZP (water) represents the ζ-potential value of the water–bentonite system and ZP (inhibitor) represents the ζ-potential value of the water–bentonite–inhibitor system.

### 4.7 FT-IR Analysis

First, 10 g of dry bentonite powder was put into the pressure chamber and compacted using 1000 psi for 30 min to prepare the bentonite wafer. Then, the bentonite wafers were soaked in water and water–inhibitor solutions for 24 h. The wet bentonite was taken out of the solutions and dried in an oven at 80 °C temperatures for approximately 16 h. After that, the dried bentonite was crushed into fine powder by pestle and mortar. The IR spectral of the powder bentonite modified with inhibitors was measured by a PerkinElmer Spectrum 400 spectrometer. The experiment was carried out at room temperature between 4000 and 400 cm^–1^.

### 4.8 Contact Angle Measurement

The contact angle measurement was conducted to check the clay’s wettability, which can determine the inhibition mechanisms of the inhibitors. First, 10 g of dry bentonite powder was placed in the pressure chamber and compacted for 30 min at 1000 psi pressure. Then, for 48 h, the bentonite wafers were immersed in water and water–inhibitor solutions. The wet bentonite wafer was removed from the solutions and dried at room temperature for approximately 72 h. To make the surfaces smooth, the wafers were again put into the pressure chamber and compacted for 5 min. Upon smoothing, a small drop of water was placed on the clay wafer’s surface and the contact angles were measured by a KRUSS Drop Shape Analyzer (DSA25).

### 4.9 COSMO-RS (Conductor-Like Screening Model for Real Solvents) Simulation

The proper explanation of the clay swelling inhibition mechanisms using experimental methods is costly and time-dependent.^44^ However, due to computer technologies’ advancement, computer simulation methods have become popular and are extremely essential for understanding the mechanisms working behind clay swelling and its inhibition systems. Computer simulations can reveal the underlying principles of swelling inhibition mechanisms by analyzing the thermodynamic properties of inhibitor–water interactions. Statistical mechanics and quantum mechanics are the two families of computer simulations, and the involved techniques are Monte Carlo (MC), molecular dynamics (MD), molecular mechanics (MM), and computational quantum chemistry (CQC) simulation. In recent times, the use of molecular dynamics simulation has gained popularity for clay stabilization studies. However, this method has some drawbacks due to its long processing times.^45^ The computer simulation COSMO-RS (COSMOtherm Version 19.0.0 (Revision 5259)) is used in this analysis to evaluate the clay swelling inhibition mechanisms of an inhibitor theoretically. The sigma surfaces and surface area (chain length) of water, and the studied ILs were investigated in this study. Furthermore, the activity coefficients and hydrogen-bond energies of the investigated ILs with water were calculated. The sigma surfaces revealed the distribution of positive and negative charges on the surfaces of water and the ILs. This charge distribution is linked to the ability of the chemicals to form hydrogen bonds, as well as their hydrophobicity and lipophilicity.^46^

## Nomenclature

BT: bentonite
BMIMCl: 1-butyl-3-methylimidazolium chloride
BMIMBF_4_: 1-butyl-3-methylimidazolium tetrafluoroborate
BMIMPF_6_: 1-butyl-3-methylimidazolium hexafluorophosphate
COSMO-RS: conductor-like screening model for real solvents
*E*_HB_: hydrogen-bond energy
FF: fracturing fluids
FT-IR: Fourier transform infrared
ILs: ionic liquids
KCl: potassium chloride
wt: weight
XRD: X-ray diffraction
